# Supplementary material for: Causal Relationship between Aging and Anorexia Nervosa: A White-Matter-Microstructure-Mediated Mendelian Randomization Analysis
Source: Biomedicines. 2024 Aug 16;12(8):1874. doi: 10.3390/biomedicines12081874 (PMC11351342; doi:10.3390/biomedicines12081874)
Supplement: Supplementary file 1 [file biomedicines-12-01874-s001.zip › Supplementary Figure/Figure S4/Singe SNP plot of MR analysis for TL to ubm-b-1534.pdf]

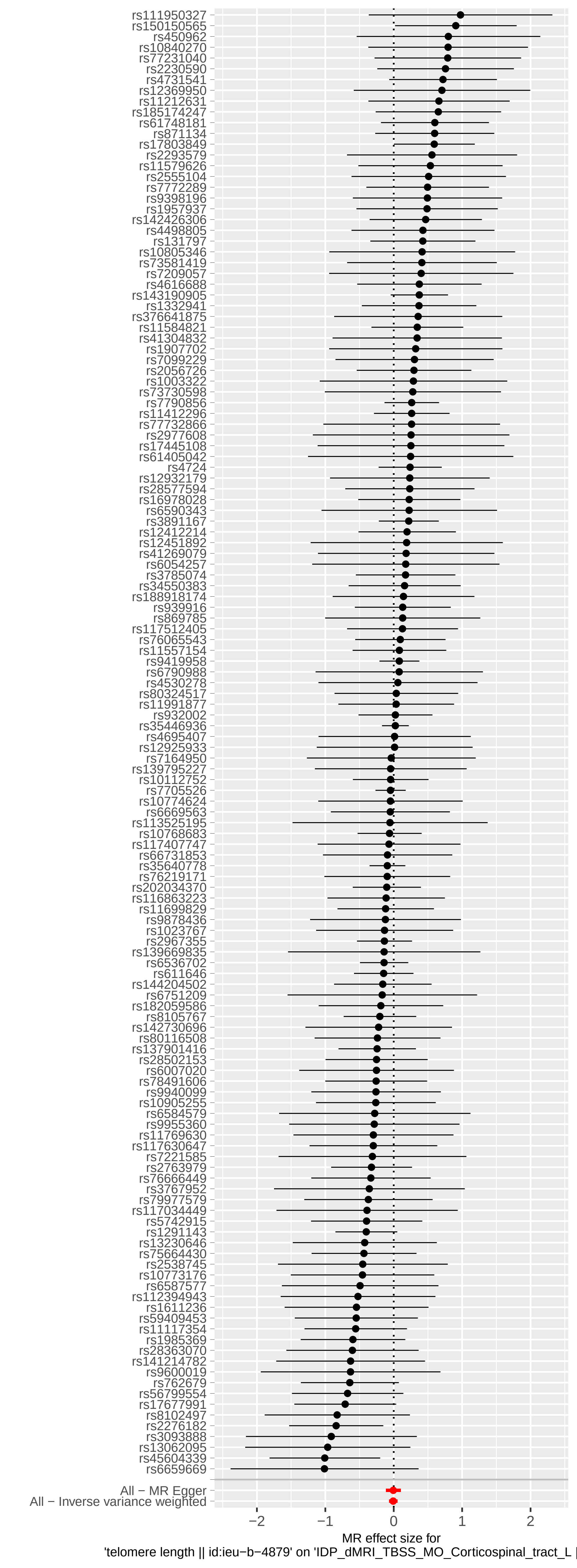
rs111950327
rs150150565
rs450962
rs10840270
rs77231040
rs2230590
rs4731541
rs12369950
rs11212631
rs185174247
rs61748181
rs871134
rs17803849
rs2293579
rs11579626
rs2555104
rs7772289
rs9398196
rs1957937
rs142426306
rs4498805
rs131797
rs10805346
rs73581419
rs7209057
rs4616688
rs143190905
rs1332941
rs376641875
rs11584821
rs41304832
rs1907702
rs7099229
rs2056726
rs1003322
rs73730598
rs7790856
rs11412296
rs77732866
rs2977608
rs17445108
rs61405042
rs4724
rs12932179
rs28577594
rs16978028
rs6590343
rs3891167
rs12412214
rs12451892
rs41269079
rs6054257
rs3785074
rs34550383
rs188918174
rs939916
rs869785
rs117512405
rs76065543
rs11557154
rs9419958
rs6790988
rs4530278
rs80324517
rs11991877
rs932002
rs35446936
rs4695407
rs12925933
rs7164950
rs139795227
rs10112752
rs7705526
rs10774624
rs6669563
rs113525195
rs10768683
rs117407747
rs66731853
rs35640778
rs76219171
rs202034370
rs116863223
rs11699829
rs9878436
rs1023767
rs2967355
rs139669835
rs6536702
rs611646
rs144204502
rs6751209
rs182059586
rs8105767
rs142730696
rs80116508
rs137901416
rs28502153
rs6007020
rs78491606
rs9940099
rs10905255
rs6584579
rs9955360
rs11769630
rs117630647
rs7221585
rs2763979
rs76666449
rs3767952
rs79977579
rs117034449
rs5742915
rs1291143
rs13230646
rs75664430
rs2538745
rs10773176
rs6587577
rs112394943
rs1611236
rs59409453
rs11117354
rs1985369
rs28363070
rs141214782
rs9600019
rs762679
rs56799554
rs17677991
rs8102497
rs2276182
rs3093888
rs13062095
rs45604339
rs6659669
All − MR Egger
All − Inverse variance weighted
−2
−1
0
1
2
MR effect size for
'telomere length || id:ieu−b−4879' on 'IDP_dMRI_TBSS_MO_Corticospinal_tract_L |
